# Cervical human papillomavirus genotypes in a high HIV setting: A scoping review of a decade of human papillomavirus epidemiological research in Botswana

**DOI:** 10.3389/fmed.2022.1020760

**Published:** 2022-11-24

**Authors:** Leabaneng Tawe, Pleasure Ramatlho, Rebecca Ketlametswe, Moses Koobotse, Erle S. Robertson, Surbhi Grover, Doreen Ramogola-Masire, Giacomo M. Paganotti

**Affiliations:** ^1^Botswana-University of Pennsylvania Partnership, Gaborone, Botswana; ^2^Office of Research and Graduate Studies, Faculty of Medicine, University of Botswana, Gaborone, Botswana; ^3^School of Allied Health Professions, University of Botswana, Gaborone, Botswana; ^4^Department of Otorhinolaryngology - Head and Neck Surgery, The Tumor Virology Program, Abramson Comprehensive Cancer Center, Perelman School of Medicine, University of Pennsylvania, Philadelphia, PA, United States; ^5^Department of Radiation Oncology, Perelman School of Medicine, University of Pennsylvania, Philadelphia, PA, United States; ^6^Department of Obstetrics and Gynecology, Faculty of Medicine, University of Botswana, Gaborone, Botswana; ^7^Division of Infectious Diseases, Perelman School of Medicine, University of Pennsylvania, Philadelphia, PA, United States; ^8^Department of Biomedical Sciences, Faculty of Medicine, University of Botswana, Gaborone, Botswana

**Keywords:** cervical human papillomavirus, genotype, high-risk types, low-risk, human immunodeficiency virus, Botswana

## Abstract

Cervical cancer burden is still high in low- and middle-income countries, including Botswana. Persistent human papillomavirus (HPV) infection is the leading cause of cervical cancer. Accurate knowledge of HPV diversity associated to cervical cancer in sub-Saharan Africa may provide accurate understanding of the natural history of HPV infection in these contexts. The goal of this review was to consolidate existing evidence on cervical HPV infection and to conduct a pooled analysis of data from all eligible Botswana studies. After a successful review of twelve studies on cervical HPV genotypes that met the inclusion criteria, HPV-16 genotype was the most frequently discovered in women with pre-cancerous and cancer lesions, followed by HPV-18. HPV-16 in HIV-positive women with precancerous lesions to cancer is between 45% and 47.7%, and between 4.5% and 26.1% for HPV-18. With reference to other HPV genotypes, the proportion of HPV-35 and HPV-58 (13-16%) seems relatively consistent among the studies, however HPV-58 appears to be more common in HIV-positive subjects compared to HIV-negative women. Indeed, HPV-45 seems to be frequently detected in women with cervical cancer compared to women with precancerous lesions. Regarding the low-risk HPV genotypes, an appropriate breakdown has been provided. In conclusion, the current prophylactic vaccines against HPV-16 and HPV-18, which have demonstrated good immunogenicity in HIV-infected populations, may still prevent infection and ultimately cancer.

## Introduction

Human papillomavirus (HPV) is the main etiological factor for cervical cancer onset and progression among women (1). However, the mere presence of the HPV does not necessarily result in cervical cancer, which demonstrates that additional co-factors acting in conjunction with HPV influence the risk of cervical cancer development. These co-factors include: HPV specific genotypes, host epigenetics, parity, behavioral factors, immunosuppression (particularly HIV-related), and other co-morbidities (2). HPV also causes cancers of the anogenital tract and head and neck, as well as genital warts and recurrent respiratory papillomatosis (3–5). Despite the introduction of screening and vaccination programmes, invasive cervical cancer (ICC) remains one of the most common malignancies in women globally (6, 7). More than 600,000 women were diagnosed with cervical cancer in 2020, and more than 340,000 are estimated to have died, worldwide (7). The incidence in low and middle-income countries is double that of high-income countries, where tremendous progress has been made in cervical cancer prevention through vaccination against HPV, as well as early detection and treatment of pre-invasive lesions (8). On the contrary, women in low and middle-income countries accounted for more than 90% of cervical cancer deaths worldwide (8). Importantly, the burden of cervical cancer in Africa is amplified by the HIV/AIDS epidemic, with HIV prevalence in sub-Saharan Africa (sSA) being among the highest in the world (8). In particular, Southern Africa has one of the world highest age standardized incidence rates of cervical cancer, being 43.1 per 100,000 on average, with respective rates: Eswatini 75.3, Lesotho 52.1, South Africa 43.5, Botswana 31.6, and Namibia at 24.2 (9). Interestingly, these incidence rates also match the world’s highest HIV prevalence recorded in these countries.

The impact of HIV on cervical cancer is demonstrated by the significantly higher incidence of cervical cancer in HIV-positive than in HIV-negative women despite antiretroviral therapy (ART) use (10). This suggests that HIV plays a critical role in cervical cancer progression. However, not all HPV infections lead to cancer. Fourteen HPV strains defined as “high-risk genotypes” have been identified by the International Agency for Research on Cancer (IARC) as either possibly or definitely carcinogenic to humans (group 1/2A carcinogens) (11). However, high-risk HPV genotypes differ substantially in their capacity to cause cancer (12). HPV-16 is the most carcinogenic of all HPV genotypes, causing more than half of all ICC worldwide (13), followed by HPV-18 and HPV-45 (12, 13), with regional variation. Indeed, the relative proportion of HPV-16 appears to be lower in sSA than other settings, while that of HPV-45 appears to be slightly greater (13–15).

In Botswana, a landlocked country in Southern Africa, cervical cancer is the leading cause of cancer death in women (6), with more than two-thirds of cases found in HIV-positive women (16). Women living with HIV are at a heightened risk of developing cervical cancer compared to women living without HIV, with data showing up to a six-fold increase in incidence compared to HIV-negative women in Botswana (17). In this context, it is critical to clarify that data on the HPV burden in general population of Botswana is still lacking (18). However, recent data on young university students (aged 18-22 years) show a rate of about 60% HPV positivity (19, 20) (Table 1). This evidence emphasizes the need to examine the prevalence of HPV among other age groups in Botswana. Botswana introduced HPV vaccine (quadrivalent) in their national immunization program in 2015 to prevent cervical and other HPV-related malignancies (21, 22). Quadrivalent HPV vaccine targeting HPV genotypes 6, 11, 16, and 18 is administered through school-based programs in a 2-dose schedule for girls without HIV infection and a 3-dose schedule for girls living with HIV (23). Botswana was the second African country to incorporate HPV vaccination into their national program, and the first to implement a two-dose immunization schedule (24).

**TABLE 1:** List of the included studies from Botswana.

Study reference	DNA extraction method	HPV diagnostic test	HPV DNA source	Age (range)	Sample size	HPV positivity rate (%)
MacLeod et al. (31)	QIAamp DNA Blood Mini Kit	Linear Array (ROCHE)	Cervical cells	29-39	139	68.3
Firnhaber et al. (30)	ND	Luminex Immuno*	Blood	24-32	170	65.9
Ramogola-Masire et al. (26)	Magna Pure	PGMY09/11(Linear array)	Cervical cells	33-39	100	92.0
Ermel et al. (27)	QIAamp MinElute Media Kit	PGMY09/11(Linear array)	FFPE biopsy	ND	171	79.5
Rantshabeng et al. (28)	Abbott	GP6 + /5 + (Abbott)	FFPE biopsy	33-44	162	82.0
Elliot et al. (32)	ND	GeneXpert	Cervical cells	40-51	103	30.0
Luckett et al. (33)	Abbott	GP6 + /5 + (Abbott)	Cervical cells	42-52	300	29.0
Tawe et al. (49)	Abbott	GP6 + /5 + (Abbott)	FFPE biopsy	29-84	126	100.0
Castle et al. (34)	ND	GeneXpert	Cervical cells	30-49	1019	99.7
McClung et al. (19)	Automated NucliSENSE EasyMag	Anyplex II HPV28 assay	Cervical cells	18-22	806	80.4
Ramatlho et al. (25)	Automated NucliSENSE EasyMag	PCR-RFLP	Cervical cells	18-20	978	60.2
Ramogola-Masire et al. (20)	Automated NucliSENSE EasyMag	Anyplex II HPV28 assay	Cervical cells	18-22	500	63.0

ND = not determined. *Non-PCR based detection.

Understanding the prevalence of the HPV genotypes in Botswana is essential for formulating HPV vaccination policy, quantifying possible impact of vaccination, and allocating resources for screening triage to prevent cervical cancer. Therefore, the purpose of this article is to examine available data on the prevalence of HPV genotypes (with or without cancer) in this peculiar context.

## Materials and methods

### Article selection

This scoping review aims to provide an overview of published articles on the prevalence of cervical HPV genotype distribution in Botswana and its relationship with cancer and HIV, when possible and/or available. Two search engines, PubMed and Google Scholar, were utilized in the procurement of widely available literature. All publications were included that matched the selection criteria “HPV” and “Botswana” published until August 2022. The titles of the references from the identified publications were also scanned for keywords matching our selection criteria and included if they met one of them. The search was repeated to identify the consistency of search terms and results. Two authors (LT and GMP) independently reviewed the titles, abstracts, and full articles of the retrieved studies.

### Study inclusion and exclusion criteria

Studies conducted in Botswana that reported the presence and prevalence of HPV genotypes were included. The inclusion was restricted to the papers published in English language until August 2022. Studies that reported HPV genotypes from anal, penile and oral sites were excluded to focus on cervical cancers HPV genotypes.

## Results

**FIGURE 1:**
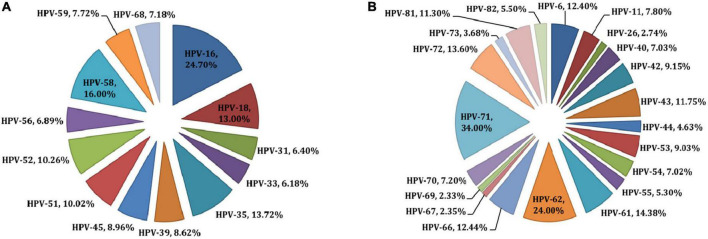
Prevalences of high-risk **(A)** and low-risk **(B)** HPV types in Botswana. All HPV types detected in individuals are reported; therefore, prevalences across all types sum to 100%.

Twelve studies from Botswana met the criteria for inclusion, with a total of 2,284 young subjects (18 to 22 years of age) who were free of cervical lesions (19, 20, 25); 559 who had cervical cancer or pre-cancer (26–29) and 1,731 who were unclassified as having any malignancies (30–34). Details on each of the included studies are presented in Tables 1, 2. Four studies (using three different cohorts), totaling 459 cervical cancer cases, examined HPV DNA from biopsies (27–29). Eight studies, totaling 3,945 subjects, examined HPV DNA from cervical cells (19, 20, 25, 26, 31–34). Finally, one study examined HPV genotypes in 170 women through a serology test (30). Table 2 shows high-risk HPV genotype-specific prevalence (HPV genotypes -16 and/or -18, alone or combined) based on the status of the cervix epithelium, from no symptoms through dysplasia to malignancy. Overall, HPV-16 genotype was the most frequently detected HPV genotype across all studies, followed by HPV-18 genotype. One important finding is that the prevalence of HPV-16 is somewhat lower in young women and increases with age. In fact, younger women are expected to have fewer exposure to risk of HPV acquisition and at the same time tend to have better immunity for clearing the virus. Furthermore, the detection of HPV-16 and/or HPV-18, alone or combined, was similar in HIV-positive and HIV-negative ICC, but HIV-positive ICC were more likely to be infected with HPV-16 than HIV-negative ICC in a study by Tawe et al. (29). Preliminary results from a small study of 30 HIV-positive women with stage 2 and 3 cervical cancers found that 50% of patients were infected with HPV-16 or HPV-18 (or both), but also 83% of women carried other high-risk genotypes (26). Regardless of the genotyping method used, the positivity rate for HPV-16 in HIV-positive women with precancerous lesions to cancer is between 45% and 47.7% (26, 27, 29), and between 4.5% and 26.1% for HPV-18 (26–29). These values are much lower (1.5% to 14%, and 0.5% to 15%, for HPV-16 and HPV-18, respectively) when young subjects are studied (19, 20, 25), and this is consistent with the natural history of HPV infection. The results of all genotyping assays were in good agreement for the HPV-16 genotype, whereas different methods showed very different results for HPV-18 genotype (Table 2). With reference to other HPV genotypes besides HPV-16 and HPV-18, the proportion of HPV-35 and HPV-58 (13-16%) (Figure 1) seems relatively common in Botswana, however HPV-58 appears to be more common in HIV-positive subjects compared to HIV-negative (2, 19, 31). Also the proportion of other high-risk HPV genotypes (HPV-39, 45, −51, −52, −56, −68) was at 7-10% (Figure 1). Indeed, HPV-45 seems to be frequently detected in women with cervical cancer compared to women with precancerous lesions (27, 35). Other high-risk HPV genotypes (HPV-31 and HPV-33) were detected at low rates (Figure 1). Regarding the low-risk HPV genotypes, the proportion of HPV-62 and HPV-71 was at 24-34%, followed by HPV-66, −72, −61, −43, and −81 at a rate of 11-14%. The rest of the low-risk HPV genotypes were detected at a proportion of less than 5% (Figure 1).

**TABLE 2:** HPV-16 and HPV-18 prevalence across the studies.

	McClung et al. (19)		Ramatlho et al. (25)	Ramogola-Masire et al. (20)	Firnhaber et al. (30)*		Castle et al. (34)		Elliot et al. (32)		Luckett et al. (33)	
HIV status	HIV +	HIV−	HIV ±	HIV ±	HIV +	HIV−	HIV +	HIV−	HIV +	HIV−	HIV +	HIV−
*N* (age range)	806 (18-22)		978 (18-20)	500 (18-22)	170 (24-32)		1,019 (30-49)		103 (40-51)		300 (42-52)	
More information	University students		University students	University students	ND		ND		ND		ND	
*−16* only	14,00%	11,00%	1.5%	8.5%	32.9%	–	8.2%	3.1%	6.0%	–	31.0%	–
*−18* only	15,00%	7,00%	0.5%	6.0%	21.8%	–	–	–	–	–	–	–
*−16* and *−18*	29,00%	18,00%	0.0%	14.2%	14.1%	–	–	–	–	–	–	–
*−16* or *−18*	–	–	2.0%	14.5%	40.6%	–	–	–	–	–	–	–

	**MacLeod et al. (31)**			**Ramogola-Masire et al. (26)**			**Rantshabeng et al.** (28)	**Ermel et al. (27)**			**Tawe et al. (49)**	

HIV status	HIV +	HIV−		HIV +	HIV−		HIV ±	HIV +	HIV−		HIV +	HIV−
*N* (age range)	139 (29-39)			100 (33-39)			162 (33-44)	171 (age N/A)			126 (29-84)	
More information	Advanced HIV and HSV-2 co-infection			Cervical lesions (CIN2 or CIN3)			Cervical lesions (HSIL)	Cervical cancer			Cervical cancer	
−*16* only	20.0%	–		45.0%	–		36.4%	45.5%	41.3%		47.7%	34.2%
−*18* only	16.8%	–		26.0%	–		4.5%	18.2%	26.1%		6.8%	7.9%
−*16* and −*18*	–	–		4.0%	–		3.8%	–	–		13.6%	15.8%
−*16* or −*18*	36.8%	–		51.0%	–		40.9%	63.7%	67.4%		54.5%	42.1%

The order of the studies is according to the cervical epithelium status (from on-cance to cancer).

*Non-PCR based detection.

The studies are organized based ion the epithelium status, from no cancer (to the left) to ICC (to the right).

## Discussion

HPV molecular studies are an important focus of research in Botswana as well as the results of the collaboration between local academics/researchers and those of the University of Pennsylvania (36). The current review evaluated the HPV genotypes among women of different age groups and HIV status (with and without cervical malignancies) to assess type-specific HPV prevalence. The data summarized in this paper represent the opportunity to describe the specific and diverse HPV genotypes in women with different risk factors for HPV acquisition. Moreover, it also provides data that will allow to possibly infer on the impact of HPV vaccination program in Botswana. In general, HPV-16 remains by far the most common high-risk HPV genotype in Botswana among women with pre-cancer and cancer, in both HIV-positive and HIV-negative. The data showed are consistent with the previously published literature investigating women living with HIV (37), but cannot fully sustain the hypothesis that HIV may substantially alters the relative carcinogenic activity of less virulent high-risk HPV genotypes as reported previously worldwide, in East Africa and in Zimbabwe (37–40). According to the available literature on Botswana, HPV-16 and HPV-18, that are known to be more prevalent in cervical cancer cases, seem to be lower in young women and increase with age and severity of the cervical lesions. The findings for HPV-16 are in agreement with those from Castellsagué et al. (41) and Edna Omar et al. (42) concerning the HPV genotypes and cervical cancer cases. However, HPV-18 shows a higher prevalence in Botswana population (26, 27, 30, 31) than in the reported studies (43, 44). Although the majority of HPV infections in early ages clear spontaneously (45, 46), some do become chronic infections and may potentially evolve to malignant transformation of the cervix.

Regarding HPV genotypes other than HPV-16 and/or HPV-18, the percentage of HPV-58 appears to be higher in HIV-positive people than HIV-negative subjects in Botswana (19, 26, 31), although these results are derived from different technical approaches, and therefore cannot be fully compared. Additionally, the most common HPV genotypes found in women with cervical cancer are HPV-45, −58 and −35 (27, 35). This is confirmed by Clifford et al. (37), where it has been shown that beside HPV-16, HPV-18, HPV-45 and HPV-58 were the more frequently genotypes detected in HIV-positive subjects with ICC compared with HIV-negative with ICC.

Concerning technical aspects of the HPV genotyping among the studies reviewed, a genotyping bias for HPV-18 could be assumed. In fact, when Abbott Real-Time High-Risk HPV platform is used, the rate of HPV-18 detection decreases significantly (Tables 1, 2). A prove of that is that when sequencing a subset of samples from Tawe et al. (29), a higher rate of HPV-18 has been found (35). However, discordance for the detection of HPV-16 or HPV-18 has been reported when different genotyping methods are applied, including the Abbott Real-Time High-Risk assay (47, 48). We therefore hypothesize that the use of the Abbott Real-Time High-Risk HPV assay may explain the low HPV-18 frequency in Tawe et al. (29) and Rantshabeng et al. (28) studies.

In summary, evidences from this review show that there is a significant presence of HPV among Botswana women, including the HPV genotypes targeted by the quadrivalent vaccine. Thus, it may be anticipated that the national HPV vaccination program in Botswana, which achieves vaccine coverage as high as 90.0%, will significantly reduce HPV burden and HPV-associated malignancies in the next few years. However, the 9-valent HPV vaccine that offers more protection against HPV than the quadrivalent HPV vaccine is highly recommended. Furthermore HPV surveillance studies should be done to monitor the evolution and prevalence of HPV infection and disease.

Important limitations of this work are that data across different studies are not always fully comparable because of differences in technical approaches for sample collection and genotyping, including the fact that the number and HPV types detected vary according to the method chosen. Additionally, HIV status is not always indicated, the severity of lesions is different and not always comparable, and ages are different across the studies. In addition, one of the papers (30) did not detect HPV DNA, instead HPV serotyping for high-risk types was performed.

In conclusion, this paper reviewed the available literature on cervical HPV genotypes in Botswana. The results highlighted that young women have a slightly lower prevalence of HPV-16, which rises with age. Independently from HIV status, cervical cancer patients have concurrently higher levels of HPV-16 and HPV-18, in Botswana. HIV-positive individuals seem to have greater HPV-58 rate than HIV-negative individuals (19, 26, 31). In addition, HPV-35 and HPV-45 is the most prevalent HPV genotype (among the non-HPV16/18 genotypes) identified in females with cervical cancer. Collectively, the currently available evidence suggests that the HPV-16 and HPV-18 based vaccines actually deployed in Botswana, may still prevent persistent HPV infection in women regardless of their HIV status, since they have demonstrated good immunogenicity in the HIV-infected population.

## Author contributions

LT and GP designed the project and prepared the tables. LT wrote the manuscript with ideas and contributions from GP. PR, RK, MK, ER, SG, and DR-M critically revised the manuscript. All authors approved the final version of the manuscript.
